# The Chronobiological and Neuroprotective Mechanisms of Resveratrol in Improving Sleep

**DOI:** 10.1155/mi/4954030

**Published:** 2025-03-19

**Authors:** Wenwen Zhu, Ailin Gong, Bin Zhang, Hanxing Cheng, Lishan Huang, Xiao Wu, Dechou Zhang, Wenbin Dai, Sen Li, Houping Xu

**Affiliations:** ^1^Geriatric Department, The Affiliated Traditional Chinese Medicine Hospital, Southwest Medical University, Luzhou, Sichuan, China; ^2^Department of Acupuncture and Tuina, The Affiliated Traditional Chinese Medicine Hospital, Southwest Medical University, Luzhou, Sichuan, China; ^3^Department of Respiratory medicine, Luzhou Longmatan District People's Hospital, Luzhou, Sichuan, China; ^4^Division of Spine Surgery, Department of Orthopedic Surgery, Nanjing Drum Tower Hospital, Affiliated Hospital of Medical School, Nanjing University, Nanjing, Jiangsu, China

**Keywords:** circadian rhythm, insomnia, mechanisms, neuroprotection, resveratrol

## Abstract

According to statistics, more than one-third of the global population currently experiences sleep problems, and about 10% of adults have been diagnosed with insomnia, a proportion that is increasing annually. Most currently used insomnia medications are not specifically developed but are discovered by chance, often resulting in unavoidable side effects like addiction. Thus, there is an urgent need to find safer and more effective therapeutic options. Resveratrol, a natural polyphenolic compound, shows significant potential in improving insomnia. Research shows that its effects may be achieved through multiple biological processes, including antiapoptosis, antioxidant activity, anti-inflammation, circadian rhythm regulation, modulation of neurotransmitters (gamma-aminobutyric acid (GABA), DA, 5-HT, cortisol), and increased levels of neurotrophic factor BDNF. Additionally, resveratrol's treatment of insomnia is closely linked to the SIRT1, AMPK, NF-*κ*B, mTOR, PI3K/Akt, and MAPK pathways. This review summarizes the mechanisms of resveratrol in treating insomnia to provide researchers with a deeper understanding of its action, which can aid in the development of novel targeted drugs and offer innovative ideas and methods for clinical insomnia treatment.

## 1. Introduction

Insomnia is a type of neurological disorder characterized by difficulty falling asleep, early waking, and frequent dreaming. Long-term insomnia patients often also experience psychological symptoms such as anxiety, depression, memory loss, and dementia [1]. According to statistics, more than one-third of the global population currently has sleep problems, and about 10% of adults have been diagnosed with insomnia [2]. Following the COVID-19 pandemic, an increasing number of people are experiencing sleep issues [3]. Insomnia often occurs alongside other diseases, acting both as a cause and a consequence. Long-term insomnia can weaken the immune system, increasing the risk of other illnesses such as diabetes and cardiovascular diseases. This results in significant economic burdens, mental stress, and health risks [4–6]. Currently, the U.S. Food and Drug Administration (FDA) has approved several medications for the treatment of insomnia. These include benzodiazepines (such as alprazolam and diazepam), benzodiazepine receptor agonists (such as eszopiclone), ramelteon (a melatonin receptor agonist), and dual orexin receptor antagonists (DORAs). Due to significant adverse effects, benzodiazepines are now rarely used for treating insomnia. Benzodiazepine receptor agonists, while having a shorter duration of action and higher safety profile compared to benzodiazepines, still have adverse effects such as next-day drowsiness, rebound insomnia, and complex sleep behaviors. Ramelteon offers higher safety than other medications, but its effectiveness in improving insomnia is not pronounced. DORAs are the latest FDA-approved medications for the treatment of insomnia in adults. They work by inhibiting wakefulness, thereby improving difficulties in falling asleep and increasing sleep duration while minimizing next-day impairment. Despite showing significant efficacy in treating insomnia and offering advantages over traditional sedative-hypnotic drugs, DORAs still have potential drawbacks and side effects. More clinical evidence is needed to establish their long-term safety [7, 8]. Overall, there are currently no safe and effective targeted treatments for insomnia.

Resveratrol is a natural polyphenolic compound primarily found in plants such as Japanese knotweed, grapes, peanuts, mulberries, raspberries, and gentian. It excels in various areas including antiaging, antioxidant, anti-inflammatory, cardiovascular protection, anticancer, neuroprotection, metabolic regulation, and mood regulation. Resveratrol is a multifunctional and safe natural compound [9]. Resveratrol holds significant potential for treating insomnia. Research indicates that resveratrol can enhance overall sleep quality, increase the duration of deep sleep, and reduce the frequency of nighttime awakenings [10]. It helps to extend total sleep time, particularly showing significant improvements in the non-rapid eye movement (NREM) sleep stage. Another study suggests that resveratrol can restore circadian rhythms in insomniac animals, enhancing their activity levels and sleep, with especially notable effects in older animals [11]. Additionally, resveratrol can help restore normal circadian rhythms and improve sleep patterns by regulating the expression of circadian clock genes in the body [12].

Resveratrol is a highly promising compound for improving insomnia. This review discusses its potential mechanisms and pathways for alleviating insomnia, which could aid in the development of new targeted therapies for insomnia and provide novel approaches and methods for clinical treatment.

## 2. Physiological Effects of Resveratrol and Its Association With Insomnia

Resveratrol is a polyphenol compound that occurs naturally in certain plants such as grapes and berries. It has a variety of properties including antioxidant and anti-inflammatory properties and is also thought to promote longevity by activating SIRT1(Table 1). Research indicates that resveratrol plays significant roles in the body, such as possessing powerful antioxidant properties that can neutralize free radicals and reduce oxidative stress [13]. It exerts anti-inflammatory effects by inhibiting the production and activity of various inflammatory mediators, such as NF-*κ*B [14]. It exhibits significant anticancer activity by inducing apoptosis (programmed cell death) in cancer cells, inhibiting cancer cell proliferation, suppressing tumor angiogenesis (the blood supply needed for tumor growth), and preventing the invasion and metastasis of cancer cells [15]. It protects the cardiovascular system by improving endothelial function, enhancing vascular elasticity and dilation, inhibiting platelet aggregation to reduce the risk of thrombosis, lowering blood pressure and cholesterol levels, and reducing low-density lipoprotein (LDL) oxidation to prevent atherosclerosis [16]. Through its antioxidant and anti-inflammatory mechanisms, resveratrol protects neural cells from damage, slows the progression of neurodegenerative diseases such as Alzheimer's and Parkinson's disease, and exhibits excellent neuroprotective effects [17, 18]. Resveratrol is believed to extend lifespan and delay the aging process primarily by activating SIRT1 (the longevity gene) and AMPK (a key regulator of energy). These pathways are associated with cellular metabolism, DNA repair, and inflammation responses [19, 20]. Resveratrol can also improve insulin sensitivity and regulate glucose and lipid metabolism, thereby helping to prevent and manage metabolic syndrome and type 2 diabetes [21, 22]. Additionally, through its antioxidant, anti-inflammatory, and metabolic regulatory functions, resveratrol can alleviate liver damage and fatty liver, thus providing liver protection [23, 24].

**Figure 1:**
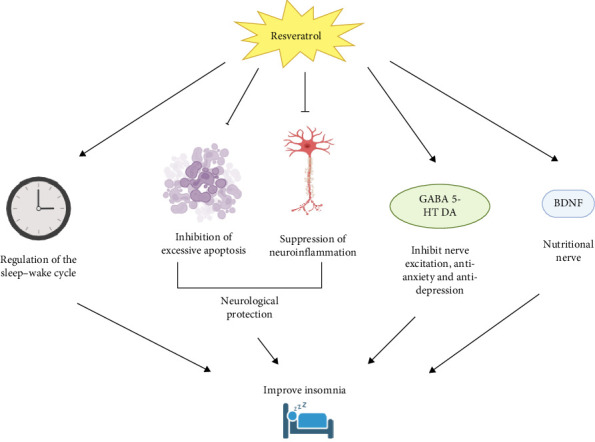
Pathways of resveratrol to improve insomnia. BDNF, brain-derived neurotrophic factor; DA, dopamine.

The relationship between resveratrol and insomnia primarily involves its effects on the nervous system and sleep regulation [25]. Research shows that resveratrol possesses strong antioxidant and anti-inflammatory properties, which help reduce oxidative damage, inflammation, and excessive apoptosis in the nervous system. This neuroprotective effect contributes to improved sleep quality [25]. Studies have shown that resveratrol can regulate the sleep–wake cycle by affecting circadian clock genes and their associated rhythms [12]. And resveratrol can modulate the sleep–wake cycle by influencing melatonin secretion and enhancing its effects [26]. Furthermore, resveratrol can regulate sleep by influencing the levels of neurotransmitters involved in sleep and mood regulation, such as GABA, serotonin (5-HT), and brain-derived neurotrophic factor (BDNF) [27, 28]. It is evident that resveratrol has excellent potential for improving insomnia (Figure 1).

## 3. Potential Mechanisms of Resveratrol in Treating Insomnia

### 3.1. Regulates Circadian Rhythms to Adjust the Sleep–Wake Cycle

Circadian clock gene regulation is essential for keeping circadian rhythms. Resveratrol has significant effects on regulating the sleep–wake cycle, primarily due to its modulation of core circadian clock genes [29]. The sleep–wake cycle, which alternates between sleep and wakefulness within a 24-hour period, is a typical example of this circadian rhythm [30, 31]. Each cycle consists of non-rapid eye movement (NREM) and rapid eye movement (REM) sleep stages, with the NREM stage gradually shortening and the REM stage gradually lengthening as the night progresses. Research has shown that REM sleep is associated with memory consolidation, emotional regulation, and cognitive function [32].

The core circadian clock gene network, consisting of CLOCK, BMAL1, PER, and CRY genes in the suprachiasmatic nucleus (SCN), plays a crucial role in the transcription-translation feedback loop (TTFL) of the biological clock. This network is essential for maintaining the sleep–wake rhythm [33]. The biological clock works through positive and negative feedback loops, which cause fluctuations in the levels of clock gene RNA and proteins, maintaining the circadian rhythm. Core circadian clock genes are divided into two groups: CLOCK and BMAL1, which promote wakefulness during the day, and PER and CRY, which induce sleep at night. These genes show distinct circadian patterns. For example, CLOCK mRNA increases in the morning, peaks during the day, and decreases at night. BMAL1 mRNA peaks in the evening and is lowest in the early morning. CRY1 and CRY2 genes peak at night, while PER1 and PER2 peak in the evening [34]. This complementary expression pattern ensures the precise operation of the biological clock and maintains the homeostatic regulation of circadian rhythms through a complex feedback regulatory network [35].

In chronic sleep deprivation experiments, the expression of Bmal1, Clock, Per, and Cry genes in mice was found to be abnormal [36], BMAL1 gene knockout in macaques exhibits reduced sleep, as well as symptoms of anxiety and depression [37]. These studies indicate that the sleep–wake cycle is closely related to the regulation of the circadian clock genome. Resveratrol plays a significant role in regulating circadian rhythms, primarily by modulating core circadian clock genes. These genes, including CLOCK, BMAL1, PER, and CRY, are key components of the biological clock that governs the sleep–wake cycle. Resveratrol influences these genes, particularly by activating SIRT1, which in turn regulates their expression to maintain circadian rhythm and improve sleep quality.

The circadian clock operates through a feedback loop involving positive and negative regulation. CLOCK and BMAL1 promote wakefulness during the day, while PER and CRY genes induce sleep at night. Resveratrol's action on SIRT1 enhances the expression of these core genes, aligning the sleep–wake cycle and promoting a healthier rhythm. This mechanism helps to address sleep disturbances, such as insomnia, by restoring the natural sleep cycle. Studies show that resveratrol's ability to regulate these genes helps synchronize the biological clock, resulting in improvements in sleep quality and duration. By fine-tuning the circadian rhythm, resveratrol contributes to the restoration of normal sleep patterns and provides relief from insomnia symptoms [29, 38].

**Figure 2:**
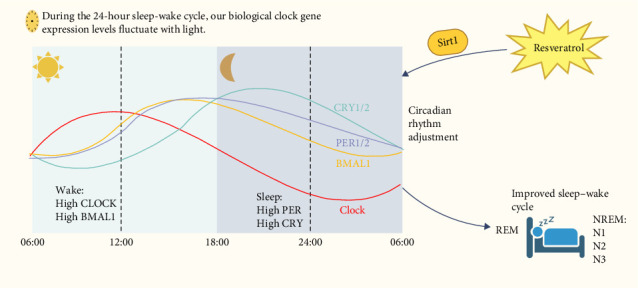
Resveratrol regulates the 24-h circadian rhythm to improve the sleep–wake cycle. High levels of CLOCK, BMAL1 maintain arousal, high levels of PER, CRY maintain sleep.Resveratrol activates SIRT1, which regulates the circadian rhythm of biological clock.

In summary, resveratrol's effects on circadian rhythms are crucial for regulating the sleep–wake cycle, making it a promising agent for improving sleep and addressing insomnia (Figure 2).

### 3.2. Antiapoptosis to Reduce Excessive Neuronal Apoptosis and Exert Neuroprotective Effects

Neural cell apoptosis is one of the significant factors leading to insomnia. Resveratrol, through its strong antiapoptotic effects, effectively reduces excessive apoptosis of neural cells and provides neuroprotective benefits [39]. Apoptosis, as one of the main forms of cell death, plays a crucial role in human growth, development, and immune cell regulation and is essential for maintaining cellular homeostasis [40]. Apoptosis can be mediated by two different pathways [41]: an intrinsic pathway, mediated by mitochondria, which mainly involves the regulation of Bcl-2 family proteins. The second is the extrinsic pathway, which is mediated by the death receptor and involves the activation of Fas-associated death structural domain protein (FADD) and Caspase 8 [42].

**Figure 3:**
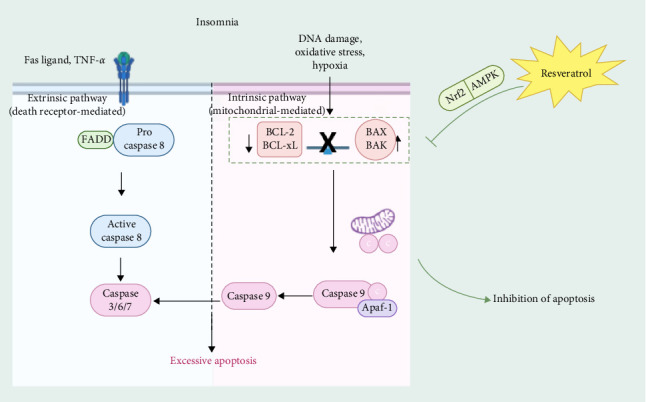
Resveratrol reduces neuronal damage associated with insomnia by inhibiting excessive cell apoptosis. Resveratrol inhibits excessive apoptosis in insomniacs by regulating the Nrf2 and AMPK pathways, e.g., by increasing the expression of the antiapoptotic. AMPK, AMP-activated protein kinase.

Studies have found that insomnia is closely related to apoptosis. Some medications used to treat insomnia have antiapoptotic effects on neurons, and insomnia can be improved by inhibiting cell apoptosis [43]. In the para-chloro-DL-phenylalanine (PCPA)-induced insomnia rat model, the expression level of the apoptosis-related B-cell lymphoma 2 gene (Bcl-2) is reduced, while the expression levels of BCL2-associated X protein (Bax), B-cell lymphoma 2 gene-associated promoter (Bad), and caspase-3 (Caspase-3) are significantly upregulated, leading to a marked increase in the extent of apoptosis [44, 45]. Research has found that resveratrol can alleviate apoptosis in lung tissues of obstructive sleep apnea (OSA) rats by activating the Nrf2/ARE pathway [46]. In another study, resveratrol was found to reverse mitochondrial damage induced by AMPK pathway inhibition and reduce myoblast apoptosis, providing a reference and foundation for research on treating OSA-hypopnea syndrome [47]. In conclusion, resveratrol has a powerful antiaging ability and can achieve a protective effect by preventing excessive apoptosis and attenuating the level of apoptosis in insomnia (Figure 3).

### 3.3. Anti-Inflammatory to Exert Neuroprotective Effects

Inflammatory response is a common pathological process in insomnia patients, with inflammation and insomnia being mutually causative. Resveratrol, through its significant anti-inflammatory effects, can alleviate or counteract neuroinflammation, providing neuroprotective benefits [48]. Inflammation is a complex response of the body to harmful stimuli, such as infections, injuries, and toxins, and is categorized into acute and chronic inflammation. Damaged cells and tissues release various inflammatory mediators, including histamines, prostaglandins, leukotrienes, cytokines (such as TNF-*α*, IL-1, and IL-6), and chemokines (such as IL-8), which trigger local vascular and cellular responses [49]. Under sustained stimulation by inflammatory mediators, acute inflammation can progress to chronic inflammation [50].

**Figure 4:**
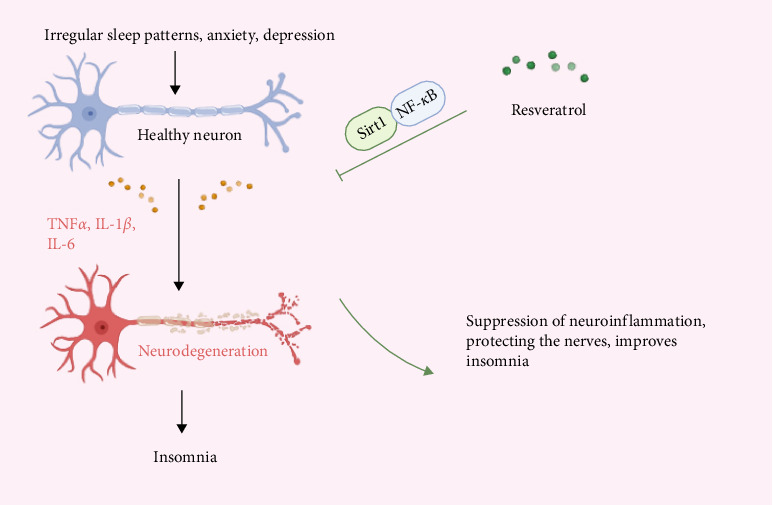
Resveratrol improves insomnia by protecting neurons through the inhibition of neuroinflammation. Long-term chronic insomnia leads to neuroinflammation. Resveratrol can inhibit neuroinflammation through SIRT1 and NF-*κ*B pathway by inhibiting inflammatory. NF-*κ*B, nuclear factor kappa B.

When inflammatory pathological changes occur in neural cells, they can cause autonomic dysfunction, disrupt sleep homeostasis, and lead to insomnia. Conversely, insomnia can activate the immune system, resulting in inflammation and other pathological changes in neural cells. In this process, the production of inflammatory mediators disrupts the delicate balance required for neural physiological functions and adversely affects memory, neural plasticity, and neural development [51]. Studies have shown that in the brain tissue of rats subjected to chronic sleep restriction, the expression of IL-1*β* and TNF-*α* is significantly increased, while the expression of BDNF is decreased [52]. In humans, acute sleep deprivation also increases the levels of proinflammatory cytokines such as IL-6 and TNF-*α* [53]. Additionally, many symptoms caused by sleep deprivation, such as drowsiness, fatigue, impaired cognitive function, and increased sensitivity to pain, can be induced by the administration of exogenous IL-1 or TNF [54, 55]. It is evident that inflammation is closely related to insomnia. Some classic sleep-regulating substances can directly or indirectly modulate inflammation [56], such as IL-1*β*, TNF-*α*, and growth hormone-releasing hormone (GHRH) for NREM sleep and prolactin and nitric oxide (NO) for REM sleep. Research indicates that resveratrol can significantly protect neural tissue and reduce anxiety and depression by activating the Sirt1/NF-*κ*B pathway to combat neuroinflammation, thereby improving sleep [57]. In another study, resveratrol improved learning and memory impairments and reduced the risk of dementia by activating Silent Information Regulator 2-related enzyme 1(SIRT1) to regulate inflammation and synaptic dysfunction [58]. In summary, resveratrol has excellent anti-inflammatory properties, which can alleviate or combat neuroinflammation, providing neuroprotective effects and thus improving symptoms of insomnia (Figure 4).

### 3.4. Regulation of GABA Inhibits Neural Hyperexcitability

GABA is a major inhibitory neurotransmitter crucial for keeping balance within the nervous system. Resveratrol enhances GABA function and inhibits excessive neuronal excitability, thus exerting sedative and hypnotic effects [59]. GABA is synthesized from glutamate through the action of glutamate decarboxylase (GAD), primarily acting on the central nervous system (CNS) to reduce neuronal excitability and regulate the balance of neural activity [60]. It has sedative and hypnotic effects, enhances memory, and possesses anti-anxiety, anti-depressant, anti-epileptic, and analgesic properties [61]. GABA has two types of receptors: the ligand-gated chloride channel GABAA receptor and the G protein-coupled GABAB receptor. When GABA binds to GABAA, chloride ions enter the cell, leading to membrane hyperpolarization and suppression of neuronal excitability [62]. When GABA binds to GABAB, it can inhibit adenylate cyclase, reduce cAMP production, inhibit the opening of calcium channels, and activate potassium channels, thereby decreasing neuronal excitability [63].

**Figure 5:**
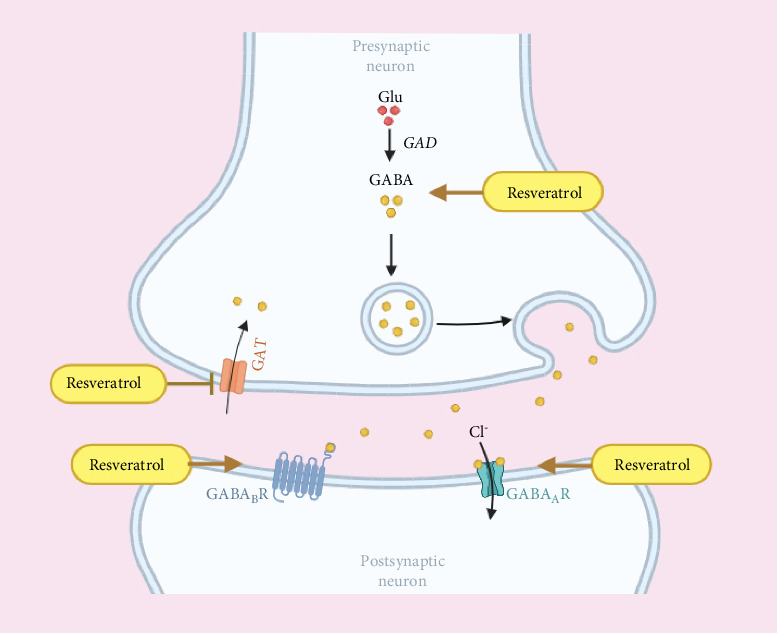
Resveratrol alleviates insomnia by regulating GABA synthesis and transport. Resveratrol promotes GABA production and GABA receptor activation, inhibits GABA reuptake, enhances GABA action, and inhibits hyperexcitability that improves insomnia.

Studies have shown that a decrease in daytime cortical GABA levels leads to a reduction in sleep drive in individuals with insomnia [64]. Activation of GABAergic neurons induces NREM sleep and inhibits REM sleep, while inhibition of GABAergic neurons leads to persistent wakefulness [65]. Another study indicates that in insomnia mice, treatment results in upregulation of GABA expression, a decrease in GLU levels, and an increase in the GABA/Glu ratio, which produces sedative and hypnotic effects, thereby improving insomnia [66]. Activation of GABAergic neurons induces NREM sleep and inhibits REM sleep, while their inhibition results in sustained wakefulness [67]. Oral GABA improves sleep onset and maintenance, reduces morning sleepiness and fatigue scores in individuals with insomnia, and is beneficial for naturally inducing sleep [68]. Research has found that resveratrol can effectively inhibit pain by activating GABA receptors in the trigeminal nerve [27], it can also exert significant anxiolytic effects by inhibiting GABA reuptake transporter 1, thereby increasing synaptic levels of GABA neurotransmitters [59]. Resveratrol can also improve Alzheimer's disease, a neurodegenerative disorder, by modulating GABA levels [69]. Additionally, resveratrol has been found to reduce excitotoxicity induced by glutamate while enhancing GABAergic inhibition, thereby lowering the glutamate/GABA ratio and exhibiting significant anticonvulsant effects [70]. In summary, resveratrol exerts significant regulatory effects on the inhibitory neurotransmitter GABA, thereby balancing excessive brain excitation in individuals with insomnia and improving sleep (Figure 5).

### 3.5. Modulation of 5-HT, Dopamine (DA) and Cortisol for Anxiolytic and Antidepressant Purposes

5-HT, DA, and cortisol are closely related to mood disorders such as anxiety and depression. Resveratrol regulates the balance of these neurotransmitters, alleviates anxiety and depressive symptoms, and further improves sleep quality [71]. Low levels of 5-HT are associated with mood disorders such as depression and anxiety [72]. Activation of 5-HT1 receptors is typically associated with wakefulness, whereas activation of 5-HT2 receptors aids in the maintenance of NREM sleep. Dysfunction of 5-HT is commonly observed in depression, and individuals with depression often experience insomnia [73]. In the prefrontal cortex, DA regulates mood, attention, and executive functions, with dysregulation being associated with disorders such as schizophrenia [74, 75]. In the CNS, DA plays a role in promoting wakefulness, with high levels of DA being associated with an alert state. DA reuptake inhibitors and DA receptor agonists can be used to treat excessive sleepiness, while DA receptor antagonists may be utilized to improve insomnia [76]. Cortisol levels rise during stress to help the body cope with pressure. It exhibits a significant circadian rhythm, with peak levels in the morning and lowest levels at night, playing a crucial role in regulating wakefulness and sleep [77]. Chronic stress and elevated cortisol levels are closely associated with insomnia. Patients with chronic insomnia often exhibit hyperactivity of the HPA axis, characterized by elevated cortisol levels at night. Medications that lower cortisol levels, such as glucocorticoid receptor antagonists, may be used to treat stress-related insomnia [78].

5-HT, DA, and cortisol play significant roles in the occurrence and maintenance of insomnia. While 5-HT and DA regulate mood, wakefulness, and sleep, cortisol influences sleep through stress responses and circadian rhythms. Research indicates that resveratrol improves depressive and anxiety-like symptoms in irritable bowel syndrome by modulating 5-HT levels [28]. Among these, increasing the expression of 5-HT can enhance brain function and alleviate depression [79]. In another study, resveratrol was found to significantly increase the levels of neurotransmitters DA and 5-HT in the prefrontal cortex, counteracting depression [80]. Additionally, resveratrol can exert neuroprotective effects and improve depressive-like behaviors by reducing cortisol release through the enhancement of brain antioxidants and monoamine levels [81]. In summary, resveratrol can significantly improve insomnia by alleviating anxiety and depressive symptoms.

### 3.6. Regulation of BDNF for Neurotrophic

BDNF is a crucial neurotrophic factor that plays a key role in neuronal survival, synaptic plasticity, neurogenesis, and various neuropsychiatric disorders [82]. BDNF exerts its effects by binding to its high-affinity receptor TrkB (tropomyosin receptor kinase B), which activates downstream signaling pathways such as PI3K/Akt, MAPK/ERK, and PLC*γ*, promoting neuronal survival and resistance to apoptosis [83]. In adult brains, BDNF enhances the proliferation and differentiation of neural stem cells in areas like the hippocampal dentate gyrus and the olfactory bulb, supporting adult neurogenesis and playing a crucial role in cognitive function and emotional regulation [84]. BDNF enhances synaptic long-term potentiation by regulating synaptic transmission efficiency and postsynaptic currents. It plays a crucial role in synapse formation and remodeling, modulating both presynaptic and postsynaptic structures, and influencing information transfer between neurons [85].

Sleep deprivation can decrease BDNF levels, affecting LTP and cognitive function. During sleep, particularly during SWS, BDNF supports synaptic plasticity and memory consolidation by regulating synaptic strength and postsynaptic signaling [86]. In patients with insomnia, reduced levels of BDNF are associated with mood disorders such as anxiety and depression. Interventions that improve sleep quality, such as cognitive behavioral therapy and pharmacological treatments, can increase BDNF levels and enhance mood and cognitive function. Numerous studies have demonstrated that resveratrol has excellent neurotrophic effects and can regulate BDNF levels in the body [80]. Resveratrol can also restore the ultrastructure of hippocampal neurons and synapses, and activate BDNF signaling pathways, thereby alleviating pain and improving cognitive impairments [87]. In another study, resveratrol was shown to increase BDNF levels, exerting neuroprotective effects and thereby improving neurodegenerative diseases [88]. In summary, resveratrol can enhance neuronal health and improve insomnia by increasing BDNF levels.

## 4. Potential Pathways of Resveratrol for the Treatment of Insomnia Disorders

**Figure 6:**
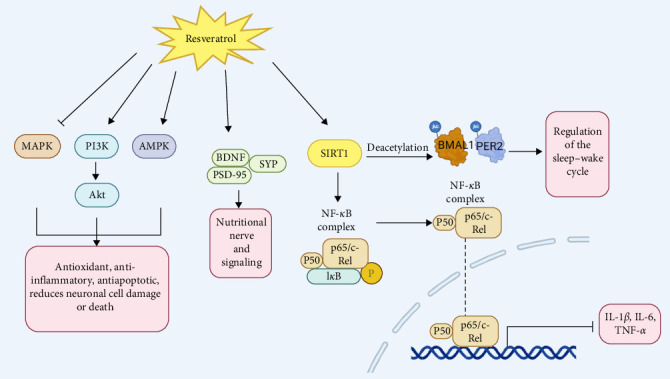
Resveratrol alleviates insomnia by regulating GABA synthesis and transport. Resveratrol promotes GABA production and GABA receptor activation, inhibits GABA reuptake, enhances GABA action, and inhibits hyperexcitability that improves insomnia. AMPK, AMP-activated protein kinase; BDNF, brain-derived neurotrophic factor; MAPK, mitogen-activated protein kinase; NF-*κ*b, nuclear factor kappa b.

Research shows that resveratrol may improve insomnia through the modulation of various signaling pathways and gene expressions, such as AMPK, SIRT1, NF-*κ*B, PI3K/Akt, and MAPK pathways [89, 90]. Notably, its impact on activating SIRT1 to regulate the expression of circadian rhythm genes is particularly prominent (Figure 6).

SIRT1 is an NAD+-dependent deacetylase involved in regulating cellular metabolism and stress responses. It has been reported that resveratrol can improve neuroinflammation as well as anxiety and depressive-like behaviors by activating the SIRT1/NF-*κ*B p65 pathway, which inhibits pro-inflammatory factors IL-1*β*, IL-6, and TNF-*α* [57, 91]. By increasing the levels of SIRT1, BDNF, Postsynaptic Density Protein 95 (PSD-95), and Synaptophysin (SYP), and by mitigating the elevation of pro-inflammatory factors, resveratrol effectively improves learning and memory impairments through the regulation of inflammation and synaptic dysfunction [58]. In addition to improving cognitive function, memory impairments, and anxiety-depressive symptoms commonly associated with sleep disorders [92], resveratrol has also been reported to regulate circadian rhythms and enhance sleep quality [93]. Research indicates that resveratrol activates SIRT1, which deacetylates BMAL1, thereby enhancing its activity and improving the amplitude of the central circadian clock [94, 95]. Additionally, resveratrol facilitates the deacetylation of PER2, leading to its degradation [93]. This affects the sleep–wake cycle and acts as a positive regulator of circadian rhythms.

AMP-activated protein kinase (AMPK) is a key regulator of cellular energy balance. Research has shown that resveratrol can activate the AMPK signaling pathway to inhibit oxidative stress, promote energy metabolism and mitochondrial function [96], and reduce neurodamage associated with insomnia, thereby improving sleep quality. Meanwhile, resveratrol promotes the phosphorylation of AMPK, activates autophagy, reduces the production of reactive oxygen species (ROS), inhibits apoptosis [90], and autophagy helps to remove damaged organelles, protects neurons from damage, and thus improves the quality of sleep. Nuclear factor kappa B (NF-*κ*B) is a critical transcription factor involved in regulating immune and inflammatory responses. Excessive inflammation is closely linked to sleep disorders. Studies have shown that resveratrol can exert anti-inflammatory effects by inhibiting abnormal activation of the NF-*κ*B signaling pathway [97], reducing the production of inflammatory factors, and thereby alleviating inflammation and improving sleep quality. The phosphoinositide 3-kinase/protein kinase B (PI3K/Akt) signaling pathway plays a crucial role in cell survival, proliferation, and metabolism. Research has shown that resveratrol can activate the PI3K/Akt pathway [98], alleviating inflammation and oxidative stress, promoting cell survival and neuroprotection, and consequently improving sleep. The mitogen-activated protein kinase (MAPK) signaling pathway plays a crucial role in cellular stress responses, proliferation, and differentiation. The research indicates that resveratrol can mitigate mitochondrial apoptosis, necroptosis, and immune dysfunction by inhibiting the MAPK signaling pathway [99]. This action reduces cellular stress and inflammation, thereby protecting neurons and improving insomnia.

In addition, it was found that resveratrol may be neuroprotective by directly acting on microglia and inhibiting their overactivation, thereby reducing inflammation and apoptosis in the brain [20].

## 5. Limitations of Resveratrol

Despite the great potential of resveratrol in improving insomnia, it is worth noting that many of the effects induced by resveratrol are dose dependent, with opposite effects occurring at low and high doses, whereby low doses may have protective effects, whereas high doses of resveratrol may have adverse effects, aggravate the onset or progression of a number of disorders, and even produce toxic effects in some cases [100]. Therefore, it is important to use resveratrol in appropriate doses. In addition, the oral bioavailability of resveratrol is low, as it is rapidly metabolised after intestinal absorption, resulting in a low effective concentration. Therefore, how to enhance its bioavailability remains a problem that needs to be addressed [101]. In addition, due to poor blood–brain barrier (BBB) permeability, resveratrol has limited penetration into the CNS. Many studies have been dedicated to addressing the issue of CNS drug delivery, such as nanoparticles (NPs) targeting multiple transport mechanisms to promote the targeted delivery of therapeutic agents to the CNS and improve therapeutic transport and efficacy across the BBB [102, 103]. Although the emergence of nanotechnology-driven drug delivery systems provides promising strategies to enhance CNS targeting and bioavailability, thus overcoming the limitations of conventional treatments, this technology has not yet been effectively applied, and related research is still limited. This continues to hinder the efficient application of resveratrol.

More epidemiological and clinical trials are needed in the future to evaluate the dose–response relationship of resveratrol in humans to further understand its potential therapeutic efficacy and safety. There is also a need for more in-depth research on nanodelivery technologies to improve the bioavailability of resveratrol.

## 6. Discussion

Insomnia is a common neurological disorder with a broad impact, posing a significant burden on individual health and socioeconomic conditions. Although existing pharmacological treatments can alleviate insomnia symptoms to some extent, their application is limited due to side effects and long-term safety concerns. Resveratrol, as a multifunctional natural polyphenolic compound, shows great potential in improving insomnia.

First, resveratrol restores normal circadian rhythms by regulating the expression of circadian clock genes [29]. Insomnia patients usually exhibit circadian rhythm disorders, and resveratrol can regulate the expression of biological clock genes BMAL1 and PER2 by activating SIRT1, thus adjusting the sleep–wake cycle and improving sleep quality. The discovery of this mechanism of action provides a new research direction for the treatment of insomnia, especially in exploring the role of natural compounds in the regulation of circadian rhythms. Second, resveratrol exerts neuroprotective effects through antiapoptotic mechanisms [104, 105]. Studies have shown that insomnia is closely related to neuronal apoptosis and cellular inflammation. Resveratrol can activate AMPK, PI3K/AKT signaling pathway, inhibit the abnormal activation of MAPK and NF-*κ*B, reduce oxidative stress, excessive apoptosis and inflammation of neuronal cells, and protect neuronal cells, thus improving the symptoms of insomnia. Resveratrol can also reduce neuroinflammation and improve sleep quality by inhibiting the production of inflammatory mediators. This mechanism reveals the potential of resveratrol in neuroprotection and suggests the prospect of resveratrol application in other inflammation-related diseases, as well as its importance in the prevention and treatment of neurodegenerative diseases. Moreover, resveratrol exerts sedative, anxiolytic, and antidepressant effects by regulating the levels of neurotransmitters such as GABA and serotonin (5-HT) [106]. GABA is the primary inhibitory neurotransmitter in the CNS, while 5-HT is closely associated with mood regulation. Resveratrol improves sleep and alleviates mood disorders by increasing GABA expression and modulating 5-HT function, thereby balancing neuronal activity in the brain. This mechanism highlights the potential of resveratrol in the treatment of mental disorders, particularly in addressing depression and anxiety.

## 7. Future Directions

In summary, resveratrol, as a multifunctional natural compound, exerts positive effects on insomnia through various mechanisms. These mechanisms include regulating circadian gene expression, antiapoptotic actions, anti-inflammatory effects, and modulation of neurotransmitter levels. Although existing studies suggest that resveratrol has significant efficacy in improving insomnia, further clinical research is needed to verify its long-term safety and effectiveness. Future studies should focus on the mechanisms of resveratrol, clinical trials, and its applicability in different populations. Comprehensive and in-depth research is essential to prove the role of resveratrol in insomnia treatment, providing safer and more effective treatment options for insomnia patients. Ultimately, we hope that resveratrol will become a breakthrough in the field of insomnia treatment, contributing to improved sleep quality and quality of life for people worldwide.

## Figures and Tables

**Table 1:** Physiological Functions of Resveratrol.

Physiological function	Pathway	References
Antioxidant	Neutralizes free radicals, reduces oxidative stress	[13]
Anti-inflammatory	Inhibits the production and release of inflammatory mediators	[14]
Anticancer	Induces apoptosis in cancer cells, inhibits cancer cell proliferation, and suppresses angiogenesis and metastasis	[15]
Cardiovascular Protection	Enhances endothelial function, regulates blood pressure and cholesterol, prevents atherosclerosis	[16]
Neuroprotection	Protects neural cells through anti-inflammatory and antioxidant mechanisms	[17, 18]
Antiaging	Activates SIRT1 (longevity gene) and AMPK (energy regulation) pathways	[19, 20]
Metabolic Regulation	Improves insulin sensitivity and regulates glucose and lipid metabolism	[21, 22]
Liver Protection	Alleviates liver damage through antioxidant, anti-inflammatory, and metabolic regulation	[23, 24]

## Data Availability

Data sharing not applicable to this article as no datasets were generated or analyzed during the current study.
